# SUMOylation of Argonaute-2 regulates RNA interference activity

**DOI:** 10.1016/j.bbrc.2015.07.073

**Published:** 2015-09-04

**Authors:** Fernando Josa-Prado, Jeremy M. Henley, Kevin A. Wilkinson

**Affiliations:** School of Biochemistry, Medical Sciences Building, University Walk, University of Bristol, Bristol, BS8 1TD, United Kingdom

**Keywords:** SUMOylation, Argonaute, PIAS3, Ubc9, RNAi

## Abstract

Post-translational modification of substrate proteins by small ubiquitin-like modifier (SUMO) regulates a vast array of cellular processes. SUMOylation occurs through three sequential enzymatic steps termed E1, E2 and E3. Substrate selection can be determined through interactions between the target protein and the SUMO E2 conjugating enzyme Ubc9 and specificity can be enhanced by substrate interactions with E3 ligase enzymes. We used the putative substrate recognition (PINIT) domain from the SUMO E3 PIAS3 as bait to identify potential SUMO substrates. One protein identified was Argonaute-2 (Ago2), which mediates RNA-induced gene silencing through binding small RNAs and promoting degradation of complimentary target mRNAs. We show that Ago2 can be SUMOylated in mammalian cells by both SUMO1 and SUMO2. SUMOylation occurs primarily at K402, and mutation of the SUMO consensus site surrounding this lysine reduces Ago2-mediated siRNA-induced silencing in a luciferase-based reporter assay. These results identify SUMOylation as a potential regulator of Ago2 activity and open new avenues for research into the mechanisms underlying the regulation of RNA-induced gene silencing.

## Highlights

• Argonaute-2 (Ago2) is SUMOylated in mammalian cells.
• SUMOylation of Ago2 takes place primarily at lysine 402.
• Abolishing Ago2 SUMOylation reduces its functional activity.

## 1 Introduction

Small RNA-mediated regulation of gene expression is a fundamental control mechanism in eukaryotic cells. RNA-induced gene silencing reduces translation of target mRNAs and is primarily mediated by members of the Argonaute (Ago) family of RNA-binding proteins [1]. There are 4 Ago proteins in humans (Ago1-4), which control RNA-induced gene silencing through binding to mRNAs complimentary to their bound RNA. Three types of RNA – microRNA (miRNA), short interfering RNA (siRNA) or PIWI-associated RNA (piRNA) – can bind to Ago family proteins [1] and silencing can occur through promoting degradation of the target transcript, inhibition of translation or, in the case of Ago2 in humans, by directly cleaving the target mRNA [2].

miRNAs are initially synthesised by RNA polymerase III and cropped in the nucleus by a processing complex consisting of the RNAse III family member Drosha and DCGR8. After processing, miRNAs are exported to the cytosol through the action of Exportin-5 and RanGTP, where they are further processed by Dicer and loaded onto Ago. This miRNA-loaded Ago is termed the RNA-induced silencing complex (RISC), and is the core effector of RNA interference (RNAi) [1].

Because of their critical role in the regulation of RNA stability and protein synthesis, Ago proteins are subject to multiple post-translational modifications (PTMs) to fine-tune their function [3]. The first reported PTM of Ago2 was prolyl 4-hydroxylation at proline 700, which increases Ago2 stability and miRNA activity during hypoxia [4], [5]. Also, hypoxia-induced phosphorylation of Y393 by EGFR reduces Ago2 association with Dicer, to promote cell survival [6]. Phosphorylation of serine 387 in Ago2 by MAPK or Akt3 regulates its localisation to cytoplasmic foci (P-bodies) enriched in RNA processing machinery [7], [8]. Furthermore, Ago2 ubiquitination regulates Ago2 turnover in activated T cells [9], while poly-ADP-ribosylation under conditions of stress recruits Ago proteins to stress granules, resulting in inhibition of their miRNA activity [10].

SUMOylation is a reversible PTM of lysine residues in substrate proteins by attachment of small ubiquitin-like modifier (SUMO) [11]. In mammals, there are three SUMO paralogues, SUMO1-3, which are conjugated to target proteins via an enzymatic pathway analogous to the ubiquitin pathway. In the nearly 20 years since its discovery, several hundred SUMO substrates have been identified which participate in nearly every cellular process. SUMOylation is particularly well-characterised for its role in the nucleus where it plays diverse roles in transcription, nuclear organisation and DNA repair [12]. Furthermore, it is becoming increasingly clear that SUMO also plays critical roles outside the nucleus, including at mitochondria and the cell surface [13], [14].

In order to be conjugated to target proteins, SUMO is first activated in an ATP-dependent manner by an E1 complex, a heterodimer of SAE1 and SAE2 in mammals, before being passed to the active site cysteine of the single SUMO conjugating enzyme, Ubc9, via a thioester bond. Ubc9 then, in conjunction with a number of known E3 enzymes, which enhance the rate and specificity of modification, transfers SUMO to the target lysine in the substrate via an isopeptide bond [11]. Importantly, SUMOylation is a reversible modification, and can be removed from substrates via the action of SUMO proteases, of which the SENP family are the best characterised [15].

The first proteins identified that possess E3 ligase activity towards SUMO were the PIAS family, which consists of 4 members, PIAS1, PIASx, PIAS3 and PIASy [16]. Each member has been shown to enhance the transfer of SUMO from thioester-loaded Ubc9 to a wide range of target proteins. The core E3 activities of the PIAS proteins require their SP-RING domains, zinc binding domains similar to those found in ubiquitin ligases of the RING-type E3 family.

By bringing together Ubc9, bound at the SP-RING domain, and SUMO, bound at the C-terminal domain, PIAS proteins enhance SUMO transfer by positioning the Ubc9 ∼ SUMO thioester optimally for substrate conjugation. In this scenario, Ubc9 primarily defines substrate specificity, since it directly binds the SUMO consensus motif, ψK×E, where ψ is a large hydrophobic residue [17]. However, for some substrates, such as the DNA sliding clamp PCNA [18], [19], direct binding of the PIAS protein to the substrate is required for efficient SUMO transfer. In these cases, a conserved domain found in all of the PIAS proteins, the PINIT domain, has been implicated in substrate binding [18], [19].

Here, using a mass spectrometry approach, we have identified Ago2 as an interactor of the PINIT domain of the SUMO E3 ligase PIAS3. Furthermore, we demonstrate that Ago2 can be SUMOylated in mammalian cells, primarily at K402, and that ablation of Ago2 SUMOylation negatively influences its RNAi activity. Together with another very recent report of Ago2 SUMOylation [20], these data provide new insight into the regulation of Ago2 function.

## 2 Materials and methods

### 2.1 Plasmid constructs

Human myc-tagged Ago2 and the firefly and renilla luciferase constructs were a gift from Dr Anna Antoniou (University of Bristol, UK). YFP-SUMO1 constructs were a gift from Prof. Frauke Melchior (ZMBH, Heidelberg, Germany). FLAG-Ubc9, and YFP-SUMO-2 constructs have been described previously [21].

### 2.2 Cell culture, transfection and lysis

Cell lines were cultured as described previously [22]. Ago2 knockout MEFs were kindly provided by Prof. Gregory Hannon (Cold Spring Harbour Laboratory, USA). For transfection of N2a cells or MEF cells, TransIT reagent (Mirus) or Lipofectamine 2000 (Invitrogen) were used, according to the manufacturers instructions. 48 h post-transfection, cells were lysed in 25 mM HEPES (pH 7.5), 150 mM NaCl, 1% Triton-X-100, 0.1% SDS and complete protease inhibitors (Roche). Cleared lysates were produced by centrifugation at 16,000 *g* for 20 min. For SUMOylation assays, cells were lysed in lysis buffer containing 20 mM NEM (*N*-Ethylmaleimide) to preserve SUMOylation. Rat brain lysate was prepared as described previously [22].

### 2.3 GST-pulldowns

GST-fusion proteins were prepared as described previously [22]. GST fusion proteins were immobilised on glutathione sepharose beads (GE Healthcare), washed 3 times with 500 μl HTG wash buffer (10 mM HEPES, 150 mM NaCl, 10% glycerol, 0.05% Triton X-100, 4 mM DTT, pH 7.5) and incubated with mammalian cell lysates (0.25–1 mg total protein) on a wheel at 4 °C for 1 h. Afterwards they were washed 3 times with 6His buffer (10 mM HEPES, 150 mM NaCl, pH 7.5) containing 0.005% Triton-X100. Proteins were eluted in Laemmli buffer, and the mixture boiled at 95 °C for 10 min. For mass spectrometry boiled samples were run on a 4–20% gradient gel (Pierce) and stained with Coomassie.

### 2.4 GFP-trap pulldowns

SUMO-1 and -2 tagged N-terminally with YFP were co-expressed with FLAG-Ubc9 and myc-Ago2 (1 μg of each for a 35 mm dish) for SUMOylation assays in N2a cells. Co-immunoprecipitations to pull-down YFP-tagged proteins were performed using GFP-trap beads (Chromotek) as described previously [21]. Final washes were carried out in 6His buffer plus 0.5% Triton-X100.

### 2.5 Mass spectrometry

Mass spectrometry was performed by Dr. Kate Heesom at the School of Biochemistry Proteomics Facility at the University of Bristol. Protein bands were cut into 1 mm^2^ pieces and digested with sequencing grade trypsin (Promega) using a ProGest automated digestion unit (Digilab Ltd.). The resulting peptides were analysed by mass spectrometry. Mass spectra were recorded in positive ion reflector mode on an Applied Biosystems 4700 MALDI mass spectrometer. Data was analyzed using the MASCOT 1.9 search engine (Matrix Science) to search against the appropriate NCBI species protein database. Search parameters allowed for one missed tryptic cleavage site, the carbamidomethylation of cysteine, and the possible oxidation of methionine; precursor ion mass tolerance was 100 ppm and fragment ion mass tolerance was 0.25 Da. All identified proteins have a Mascot score greater than 95% (the default MASCOT threshold for such searches), corresponding to a statistically significant (p < 0.05) identification.

### 2.6 Western blotting

Western blotting was performed as described previously [22]). Antibodies used were: anti-myc-tag (Cell Signalling Technology 2276 (9B11)), anti-Ago2 (Cell Signalling Technology, #2897 (C34C6)), anti-GFP (Santa Cruz Biotech. sc-8334), anti-actin (Sigma–Aldrich A5441). HRP-conjugated secondary antibodies were obtained from Sigma.

### 2.7 Ago2 functional assays

Ago2 null MEFs were co-transfected with 1 μg of myc-tagged WT hAgo2, or its mutants (K402R or 3mutCS) or GFP, 0.2 μg of Renilla luciferase (RL), 0.2 μg of Firefly luciferase (FL) and 30 pmoles of siRNA against RL or a scrambled siRNA, using Lipofectamine 2000. Cells were lysed and the reporter activities from RL and FL were measured using the Dual-Luciferase Reporter Assay System (Promega) and a Fluoroskan Ascent FL microplate fluorometer and luminometer equipped with dispensers (Thermo Fisher Scientific). RL signal was then normalised to FL signal.

### 2.8 Statistical analysis

All quantified results shown are the mean ± SEM. Statistical analyses were performed using GraphPad Prism. Differences between data sets were assessed by one-way ANOVA with Newman–Keuls multiple comparison *post-hoc* test.

## 3 Results

### 3.1 Ago2 interacts with the PINIT domain of PIAS3

**Fig. 1:**
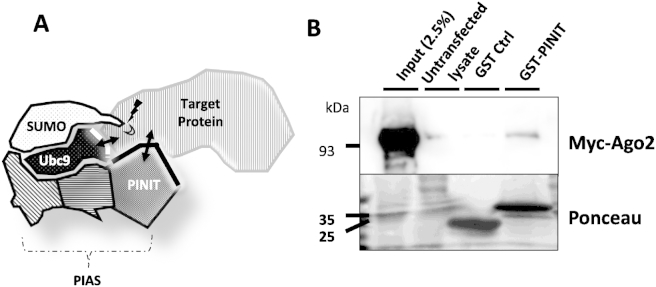
Ago2 interacts with the PINIT domain of PIAS3. A) Schematic of the proposed function of PIAS proteins in regulating SUMO modification. PIAS proteins bind Ubc9 and SUMO, positioning them in an orientation conducive to SUMO transfer, but can also directly contact substrates through the PINIT domain. B) Myc-Ago2 binds GST-PIAS3-PINIT. Myc-Ago2 was transfected into COS-7 cells, and lysates subjected to GST pulldown with GST or GST-PIAS3-PINIT followed by western blotting for the myc-tag in Ago2. The Ponceau staining reflects the amount of GST control or GST-PINIT used for the pulldowns.

To identify putative SUMO target proteins, we fused the PINIT domain of the SUMO E3 ligase PIAS3 to GST (Fig. 1A). After purification of GST-PINIT from bacteria we used this fusion protein as a bait to pulldown interacting proteins from both COS-7 cell lysate and whole rat brain homogenate and subjected the captured proteins to mass spectrometry analysis.

Several putative PIAS3-PINIT interacting proteins, including the presynaptic active zone proteins CAST1 and CAST2 and the neuronal type III intermediate filament protein peripherin (data not shown) were detected from brain lysate. Interestingly, we also identified Ago2 as a GST-PINIT interactor from both brain homogenate and COS-7 cells. Because of the fundamental role of Ago2 and the potential importance of its regulation by SUMOylation we focused on Ago2 as a novel SUMO substrate. We validated the interaction between GST-PINIT and Ago2 using GST pulldowns. Lysates from COS-7 cells transfected with N-terminally myc-tagged Ago2 were incubated with glutathione beads loaded with GST or GST-PINIT. As expected GST-PINIT, but not GST alone, pulled down myc-Ago2 (Fig. 1B). These data suggest that Ago2 may be a novel SUMO substrate.

### 3.2 Ago2 is SUMOylated in mammalian cells

**Fig. 2:**
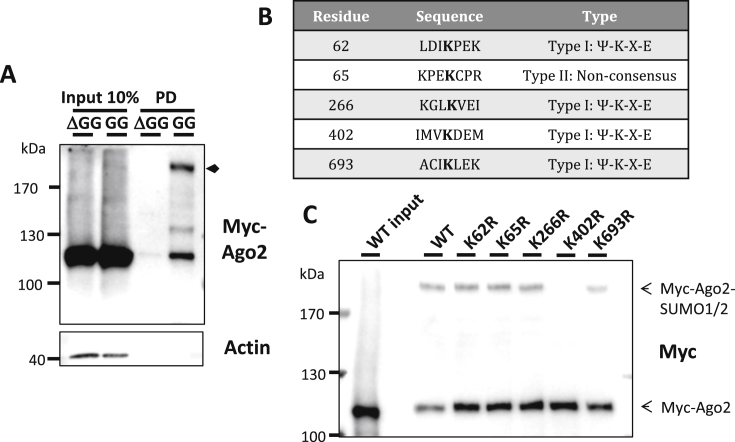
Ago2 is SUMOylated in mammalian cells, primarily at K402. A) Myc-Ago2 was transfected into N2a cells with FLAG-Ubc9 and either conjugatable (GG) or non-conjugatable (ΔGG) forms of YFP-SUMO1 and YFP-SUMO2. SUMOylated proteins were enriched on GFP-trap beads followed by western blotting for myc. The putative Ago-SUMO band is indicated by an arrow. B) Table demonstrating putative SUMOylation sites in human Ago2 as determined by the web-based SUMOylation prediction program SUMOsp. C) Mutation of K402 to arginine largely abolishes Ago2 SUMOylation. N2a cells were transfected as in A) with wild-type or various point-mutants of myc-Ago2, followed by enrichment of SUMOylated species on GFP-trap beads and western blotting for myc.

We next transfected N2a neuroblastoma cells with myc-Ago2, the SUMO conjugating enzyme Ubc9, and conjugatable or non-conjugatable (lacking the C-terminal diglycine motif required for conjugation) YFP-tagged SUMO1 and SUMO2. SUMOylated proteins were enriched by pulldown with GFP-trap beads, followed by western blotting for myc (Fig. 2A). Consistent with SUMO modification of Ago2, we detected a higher molecular weight form of Ago2 only in the presence of conjugatable SUMO. Interestingly, in the presence of conjugatable SUMO we also consistently observed stronger binding of non-modified Ago2 to the GFP-trap beads (compared to the non-conjugatable SUMO), suggesting Ago2 may additionally interact non-covalently with another SUMOylated protein.

### 3.3 Ago2 is SUMOylated primarily at K402

Analysis of the human Ago2 protein sequence using the SUMOylation site prediction software SUMOsp revealed 5 potential high-probability SUMO target lysines – K62, K65, K266, K402 and K693 (Fig. 2B). We therefore mutated each of these lysines to arginine in myc-Ago2, and examined whether these mutants could be SUMOylated in mammalian cells. As previously, N2a cells were transfected with myc-Ago2, FLAG-tagged Ubc9, and YFP-SUMO1 and YFP-SUMO2 and YFP-SUMO-modified species enriched using GFP-trap beads. Ago2-SUMOylation was unaffected by mutation of K62, 65, 266 or 693 to arginine, but was very strongly reduced upon mutation of K402 (Fig. 2C), suggesting that Ago2 is primarily modified by SUMO at K402, which lies within the SUMOylation consensus sequence ^401^VKDE.

### 3.4 Ago2 is SUMOylated by both SUMO1 and SUMO2

**Fig. 3:**
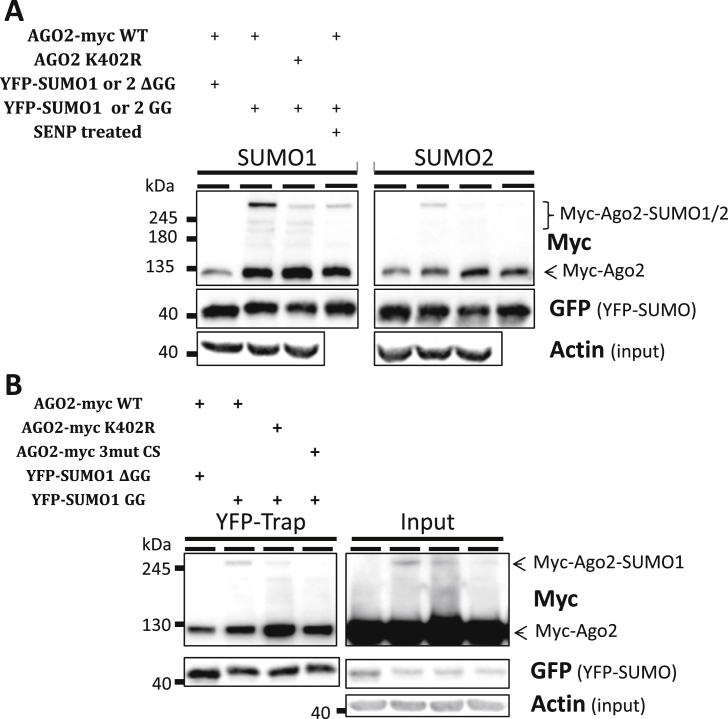
Mutation of the SUMOylation consensus site in Ago2 abolishes SUMOylation. A) Myc-Ago2 can be modified at K402 by both SUMO1 and SUMO2. N2a cells were transfected as indicated, and SUMOylated proteins enriched on GFP-trap beads followed by western blotting. For one set, after the pulldown and extensive washing, the beads were treated with the recombinant catalytic domain of the SUMO-specific protease SENP1 to further confirm the identity of the higher molecular weight species of Ago2 as Ago2-SUMO. B) Ablation of the SUMOylation consensus site surrounding K402 reduces the residual SUMOylation observed upon mutation of K402 to alanine. Cells were transfected as indicated, followed by enrichment of YFP-SUMOylated proteins on GFP-trap beads and western blotting.

Since the higher molecular weight form of Ago2 identified in Fig. 2A and C could represent SUMO1 or SUMO2 modification of Ago2, we next determined which SUMO paralogues modify Ago2. N2a cells were transfected with myc-Ago2 WT or K402R, FLAG-Ubc9 and either YFP-SUMO1 or YFP-SUMO2 in either their conjugatable or non-conjugatable (ΔGG) forms. Proteins conjugated to YFP-SUMO were isolated using GFP-trap beads and higher molecular weight forms of Ago2 were visible with the conjugatable forms of both SUMO1 and SUMO2, suggesting Ago2 can be modified by both paralogues (Fig. 3A). Furthermore, mutation of K402 dramatically reduced SUMOylation, indicating that K402 is the primary site of modification by both SUMO1 and SUMO2. We noted, however, that some residual modification of Ago2 with SUMO1 remained in the K402 point mutant (Fig. 3A). To further confirm that the higher molecular weight band observed in these assays is SUMOylated Ago2, we treated one set of pulldowns with the recombinant catalytic domain of the SUMO-specific deSUMOylating enzyme SENP1. As expected, SENP1 reduced the higher molecular weight band to a level similar to mutation of K402 (Fig. 3A). Taken together, these data indicate that Ago2 can be modified by both SUMO1 and SUMO2, primarily at K402.

### 3.5 Mutation of the SUMOylation consensus site at K402 ablates Ago2 SUMOylation

Because some Ago2 SUMOylation persisted in the K402 mutant we wondered if Ago2 is modified at other lysines when the preferred K402 is no longer available. However, the observation that mutation of the other high-probability lysines did not reduce Ago2 SUMOylation (Fig. 2C) suggests that these sites are not strongly recognised by the SUMOylation machinery. SUMOylation within a consensus site proceeds via recognition of this motif by the SUMO conjugating enzyme Ubc9. Intriguingly, however, Ubc9 can still recognise a SUMOylation consensus motif in which the target lysine has been mutated to arginine [17]. Therefore, we investigated if Ubc9 still recognises Ago2 within the SUMOylation consensus site after K402 has been mutated, but now covalently modifies adjacent lysine residues. We generated a mutant in which the consensus motif was changed from ^401^VKDE to ^401^AKAA (Ago2-3mutCS). We then transfected myc-Ago2 WT, K402R or 3mutCS into N2a cells with FLAG-Ubc9 and conjugatable or non-conjugatable SUMO1. Interestingly, despite retaining lysine 402, mutation of the SUMOylation consensus site in the 3mutCS mutant reduced SUMOylation of Ago2 more profoundly than mutation of K402 to arginine, strongly suggesting that the residual SUMOylation of the K402R mutant is mediated through recruitment of Ubc9 via the consensus sequence surrounding K402 (Fig. 3B).

### 3.6 SUMOylation of Ago2 regulates its RNAi activity

**Fig. 4:**
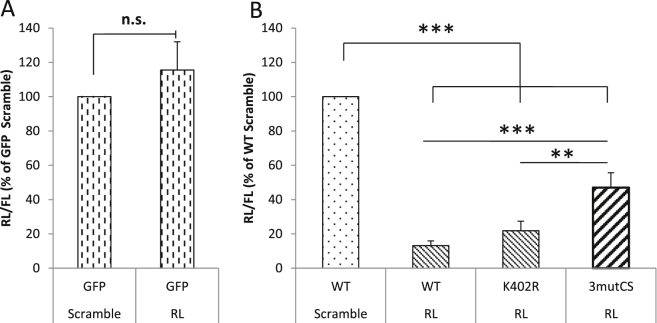
SUMOylation of Ago2 is required for correct siRNA activity. Ago2 knockout MEFs were transfected with plasmids encoding firefly luciferase, renilla luciferase, and either a scrambled siRNA or renilla luciferase siRNA along with GFP (A) or wild-type or mutant Ago2 (B). 48 h post-transfection, cells were lysed and luciferase activity quantified. Renilla luciferase levels were normalised to firefly luciferase (n = 4; ** = p < 0.01, *** = p < 0.001).

The best-characterised role of Ago2 is the processing of microRNAs to mediate degradation of target mRNAs. We therefore examined the role of Ago2 SUMOylation on this process. Since Ago2 is solely responsible for the processing of siRNAs [23], we examined siRNA efficiency as readout of Ago2 function. To exclude any confounding effect of endogenous Ago2, we performed these assays in Ago2 knockout MEFs. We transfected MEFs with renilla luciferase, firefly luciferase and either a scrambled siRNA, or an siRNA targeting renilla luciferase. Levels of renilla and firefly luciferase were then measured with a luminometer, and renilla luciferase levels normalised to firefly luciferase. Under control conditions, where GFP was transfected instead of Ago2, the siRNA against renilla luciferase had no effect (115.5% ± 16.4%), confirming the requirement of Ago2 for siRNA activity in the knockout MEFs (Fig. 4A). Expression of myc-Ago2 WT dramatically reduced renilla luciferase levels (13.1% ± 2.8%; Fig. 4B). Surprisingly, the Ago2 K402 mutant was as efficient as WT Ago2 in mediating knockdown of renilla luciferase (21.8% ± 5.6%). However, knockdown of renilla luciferase was significantly attenuated (47.0% ± 8.6%) in the Ago2 3mutCS mutant, in which the docking site for Ubc9 was ablated. Taken together, these results suggest that SUMOylation of Ago2 may be required for its full RNAi activity.

## 4 Discussion

Despite the fact that RNA interference is now well-established as a fundamental gene regulatory mechanism in eukaryotic cells, many key questions remain about how this pathway is regulated. Here, we show that Ago2, the core catalytic component of the RNAi pathway, is a substrate for SUMOylation in our ectopic expression system. Ago2 was identified as an interactor of the PINIT domain of PIAS3 and, in mammalian cell SUMOylation assays, is SUMOylated by both SUMO1 and SUMO2.

Ago2 is primarily SUMOylated at K402, although some residual SUMOylation of Ago2 occurs when this lysine is mutated to an arginine. Importantly, this remaining SUMOylation was almost completely abolished by mutation of the SUMOylation consensus site surrounding K402. These results suggest that the consensus motif represents the major binding site for the SUMO conjugating enzyme Ubc9 and that, when lysine 402 is not available for modification, Ubc9 can instead modify nearby lysines. Indeed, analysis of the structure of Ago2 reveals several lysines (K39, K52, K212 and K739) that lie in close proximity to K402, and may represent SUMOylation targets in the absence of K402 (Supp. Fig. 1). Importantly, disruption of Ubc9 recruitment by mutation of the consensus site surrounding K402 site led to a significant reduction in the RNAi activity of Ago2, highlighting the potential role of SUMOylation in regulating Ago2 function.

While this manuscript was in preparation, work from the Dejean lab has independently identified Ago2 as a substrate for SUMOylation [20]. Consistent with our results, Sahin et al. (2014) identified K402 as the major SUMOylation site. Moreover, similar to our results, they also observed some residual SUMOylation upon mutation of K402 to arginine and observed no effect of mutating K402 on the RNAi activity of Ago2. Importantly, however, we did observe an effect of mutation of the SUMOylation consensus site surrounding K402 on Ago2-dependent siRNA activity. While we cannot exclude the possibility that the effect of the 3mutCS mutant occurs independently from SUMOylation, our results indicate an effect of Ago2 SUMOylation on the efficiency of siRNA-mediated mRNA processing. Taken together, these data suggest that the residual SUMOylation of Ago2 observed for the K402R mutant is sufficient to maintain RNAi activity of Ago2. Preventing this residual SUMOylation by removing the consensus Ubc9 binding motif reveals a requirement for Ago2 SUMOylation on RNAi activity (Supp. Fig. 2). Sahin et al. (2014) further reported a role for Ago2 SUMOylation in protecting Ago2 from degradation. Mutation of K402 to arginine, or depletion of Ubc9, increased the stability of Ago2, suggesting that SUMOylation may play a multifaceted role in Ago2 function and stability [7].

Exactly how SUMOylation of Ago2 regulates RNAi activity is currently unclear. K402 lies close to the surface in Ago2 that mediates binding to Dicer, suggesting that mutation of the SUMOylation consensus sequence or addition of a ∼10 kDa SUMO protein to this site may regulate the ability of Ago2 to undergo RNA loading by Dicer. Furthermore, phosphorylation of Ago2 at Y393, nine residues upstream of the SUMOylated lysine, has been reported to occur during hypoxia and reduce binding to Dicer [6]. Given the growing number of examples of cross-talk between phosphorylation and SUMOylation pathways, it seems possible that phosphorylation of Y393 promotes Ubc9 recruitment to the consensus site surrounding K402 to enhance Ago2 SUMOylation and regulate RISC complex assembly under conditions of hypoxia. Consistent with this, Sahin et al. (2014) observed reduced SUMOylation of Ago2 upon mutation of Y393 to alanine [20]. Indeed, this model is particularly attractive given the massive increase in protein SUMOylation upon hypoxia induction, and suggests one of these target proteins may be Ago2.

In summary, we have identified Ago2 as a novel substrate for SUMOylation and demonstrate a potential role for Ago2 SUMOylation in regulating its RNAi activity. These data add to the ever-growing diversity of cellular processes known to be regulated by SUMOylation, and enhance our knowledge of the complex regulatory mechanisms that fine tune Ago2-mediated control of gene expression in eukaryotic cells.

## References

[1] Wilson R.C., Doudna J.A. (2013). Molecular mechanisms of RNA interference. Annu. Rev. Biophys..

[2] Meister G. (2013). Argonaute proteins: functional insights and emerging roles. Nat. Rev. Genet..

[3] Jee D., Lai E.C. (2014). Alteration of miRNA activity via context-specific modifications of Argonaute proteins. Trends Cell Biol..

[4] Qi H.H., Ongusaha P.P., Myllyharju J., Cheng D., Pakkanen O., Shi Y., Lee S.W., Peng J., Shi Y. (2008). Prolyl 4-hydroxylation regulates Argonaute 2 stability. Nature.

[5] Wu C., So J., Davis-Dusenbery B.N., Qi H.H., Bloch D.B., Shi Y., Lagna G., Hata A. (2011). Hypoxia potentiates microRNA-mediated gene silencing through posttranslational modification of Argonaute2. Mol. Cell. Biol..

[6] Shen J., Xia W., Khotskaya Y.B., Huo L., Nakanishi K., Lim S.O., Du Y., Wang Y., Chang W.C., Chen C.H., Hsu J.L., Wu Y., Lam Y.C., James B.P., Liu X., Liu C.G., Patel D.J., Hung M.C. (2013). EGFR modulates microRNA maturation in response to hypoxia through phosphorylation of AGO2. Nature.

[7] Horman S.R., Janas M.M., Litterst C., Wang B., MacRae I.J., Sever M.J., Morrissey D.V., Graves P., Luo B., Umesalma S., Qi H.H., Miraglia L.J., Novina C.D., Orth A.P. (2013). Akt-mediated phosphorylation of argonaute 2 downregulates cleavage and upregulates translational repression of MicroRNA targets. Mol. Cell.

[8] Zeng Y., Sankala H., Zhang X., Graves P.R. (2008). Phosphorylation of Argonaute 2 at serine-387 facilitates its localization to processing bodies. Biochem. J..

[9] Bronevetsky Y., Villarino A.V., Eisley C.J., Barbeau R., Barczak A.J., Heinz G.A., Kremmer E., Heissmeyer V., McManus M.T., Erle D.J., Rao A., Ansel K.M. (2013). T cell activation induces proteasomal degradation of Argonaute and rapid remodeling of the microRNA repertoire. J. Exp. Med..

[10] Leung A.K., Vyas S., Rood J.E., Bhutkar A., Sharp P.A., Chang P. (2011). Poly(ADP-ribose) regulates stress responses and microRNA activity in the cytoplasm. Mol. Cell.

[11] Wilkinson K.A., Henley J.M. (2010). Mechanisms, regulation and consequences of protein SUMOylation. Biochem. J..

[12] Jentsch S., Psakhye I. (2013). Control of nuclear activities by substrate-selective and protein-group SUMOylation. Annu. Rev. Genet..

[13] Geiss-Friedlander R., Melchior F. (2007). Concepts in sumoylation: a decade on. Nat. Rev. Mol. Cell Biol..

[14] Martin S., Wilkinson K.A., Nishimune A., Henley J.M. (2007). Emerging extranuclear roles of protein SUMOylation in neuronal function and dysfunction. Nat. Rev. Neurosci..

[15] Hickey C.M., Wilson N.R., Hochstrasser M. (2012). Function and regulation of SUMO proteases. Nat. Rev. Mol. Cell Biol..

[16] Rytinki M.M., Kaikkonen S., Pehkonen P., Jaaskelainen T., Palvimo J.J. (2009). PIAS proteins: pleiotropic interactors associated with SUMO. Cell. Mol. Life Sci..

[17] Sampson D.A., Wang M., Matunis M.J. (2001). The small ubiquitin-like modifier-1 (SUMO-1) consensus sequence mediates Ubc9 binding and is essential for SUMO-1 modification. J. Biol. Chem..

[18] Reindle A., Belichenko I., Bylebyl G.R., Chen X.L., Gandhi N., Johnson E.S. (2006). Multiple domains in Siz SUMO ligases contribute to substrate selectivity. J. Cell Sci..

[19] Yunus A.A., Lima C.D. (2009). Structure of the Siz/PIAS SUMO E3 ligase Siz1 and determinants required for SUMO modification of PCNA. Mol. Cell.

[20] Sahin U., Lapaquette P., Andrieux A., Faure G., Dejean A. (2014). Sumoylation of human argonaute 2 at lysine-402 regulates its stability. PLoS One.

[21] Kantamneni S., Wilkinson K.A., Jaafari N., Ashikaga E., Rocca D., Rubin P., Jacobs S.C., Nishimune A., Henley J.M. (2011). Activity-dependent SUMOylation of the brain-specific scaffolding protein GISP. Biochem. Biophys. Res. Commun..

[22] Berndt A., Wilkinson K.A., Heimann M.J., Bishop P., Henley J.M. (2013). In vivo characterization of the properties of SUMO1-specific monobodies. Biochem. J..

[23] Liu J., Carmell M., Rivas F., Marsden C., Thomson J., Song J., Hammond S., Joshua-Tor L., Hannon G. (2004). Argonaute2 is the catalytic engine of mammalian RNAi. Science.

