# Supplementary material for: SUMOylation of Argonaute-2 regulates RNA interference activity
Source: Biochem Biophys Res Commun. 2015 Sep 4;464(4):1066–71. doi: 10.1016/j.bbrc.2015.07.073 (PMC4624959; doi:10.1016/j.bbrc.2015.07.073)
Supplement: Supplementary file 1 [file mmc1.pptx]

## Slide 1
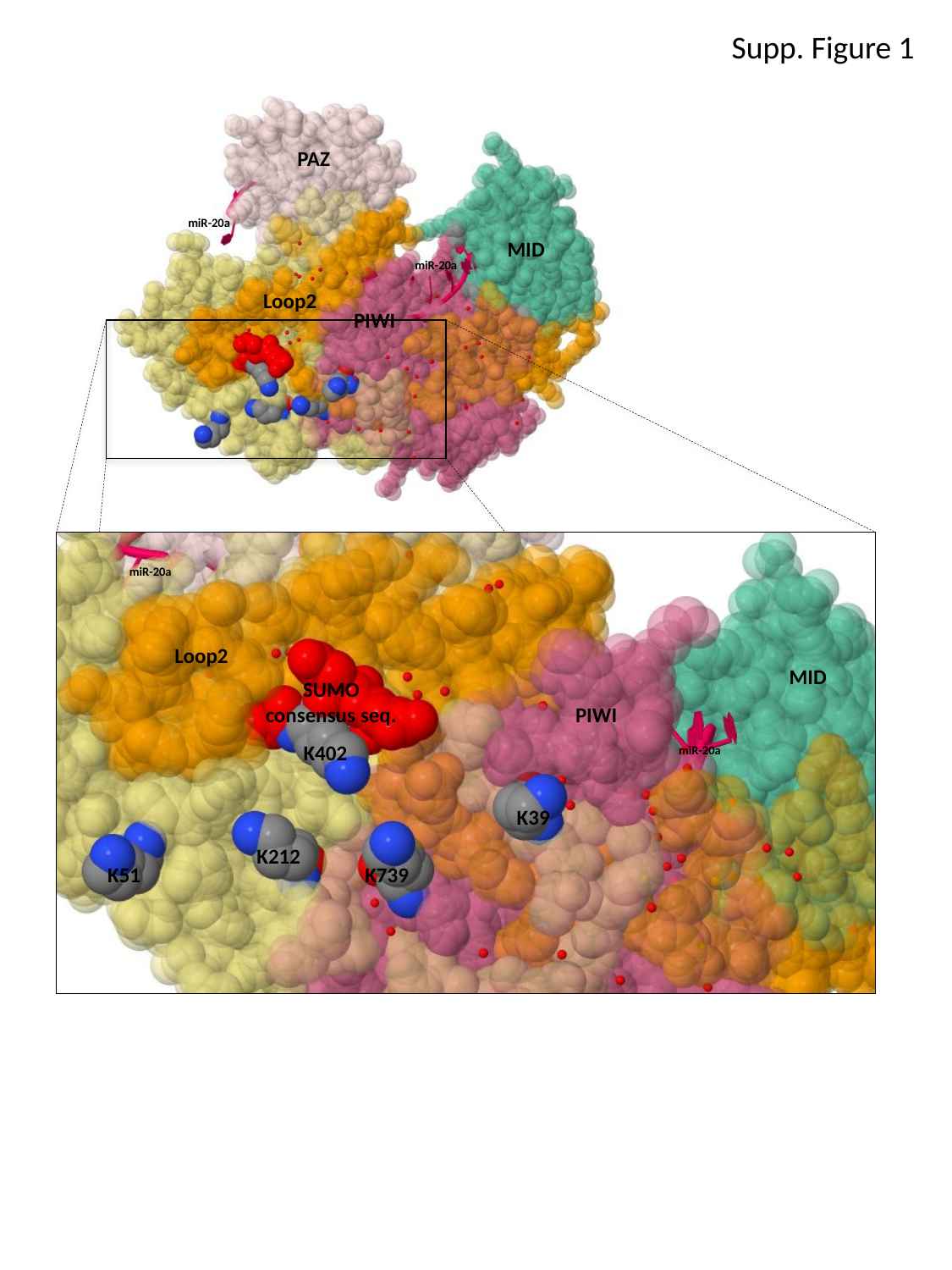

Supp. Figure 1
PAZ
miR-20a
MID
miR-20a
Loop2
PIWI
miR-20a
Loop2
MID
SUMO consensus seq.
PIWI
K402
miR-20a
K39
K212
K51
K739

## Slide 2
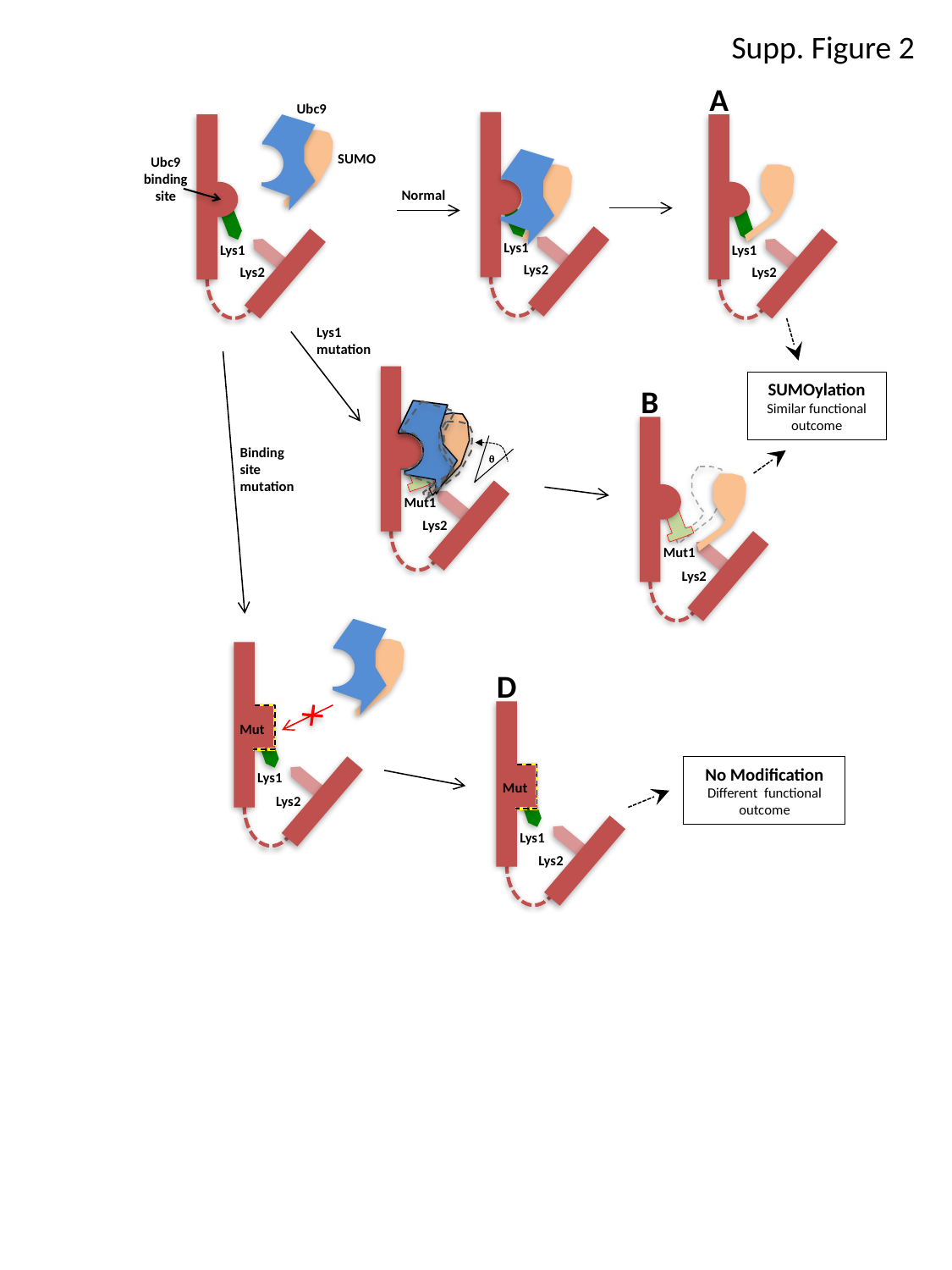

Supp. Figure 2
A
Ubc9
SUMO
Ubc9 binding site
Normal
Lys1
Lys1
Lys1
Lys2
Lys2
Lys2
Lys1 mutation
SUMOylation
Similar functional outcome
B
q
Binding site mutation
Mut1
Lys2
Mut1
Lys2
D
Mut
No Modification
Different functional outcome
Lys1
Mut
Lys2
Lys1
Lys2
